# Impairment of NADH dehydrogenase and regulation of anaerobic metabolism by the small RNA RyhB and NadE for improved biohydrogen production in *Enterobacter aerogenes*

**DOI:** 10.1186/s13068-017-0938-2

**Published:** 2017-10-30

**Authors:** Yan Wu, Yaqiao Hao, Xuan Wei, Qi Shen, Xuanwei Ding, Liyan Wang, Hongxin Zhao, Yuan Lu

**Affiliations:** 1 Zhejiang Province Key Laboratory of Plant Secondary Metabolism and Regulation, College of Life Sciences, Zhejiang Sci-Tech University, Hangzhou, 310018 China; 2 Key Lab of Industrial Biocatalysis, Ministry of Education, Department of Chemical Engineering, Tsinghua University, Beijing, 100084 China; 3 Department of Chemical Engineering, Institute of Biochemical Engineering, Tsinghua University, Beijing, 100084 China; 4 College of Life Science, Shenyang Normal University, Shenyang, 110034 China

**Keywords:** *Enterobacter aerogenes*, Biohydrogen, NADH dehydrogenase, CRISPR-Cas9, Small RNA RyhB

## Abstract

**Background:**

*Enterobacter aerogenes* is a facultative anaerobe and is one of the most widely studied bacterial strains because of its ability to use a variety of substrates, to produce hydrogen at a high rate, and its high growth rate during dark fermentation. However, the rate of hydrogen production has not been optimized. In this present study, three strategies to improve hydrogen production in *E. aerogenes*, namely the disruption of *nuoCDE*, overexpression of the small RNA RyhB and of NadE to regulate global anaerobic metabolism, and the redistribution of metabolic flux. The goal of this study was to clarify the effect of *nuoCDE*, RyhB, and NadE on hydrogen production and how the perturbation of NADH influences the yield of hydrogen gas from *E. aerogenes*.

**Results:**

NADH dehydrogenase activity was impaired by knocking out *nuoCD* or *nuoCDE* in *E. aerogenes* IAM1183 using the CRISPR-Cas9 system to explore the consequent effect on hydrogen production. The hydrogen yields from IAM1183-CD(*∆nuoC*/*∆nuoD*) and IAM1183-CDE (*∆nuoC*/*∆nuoD*/*∆nuoE*) increased, respectively, by 24.5 and 45.6% in batch culture (100 mL serum bottles). The hydrogen produced via the NADH pathway increased significantly in IAM1183-CDE, suggesting that *nuoE* plays an important role in regulating NADH concentration in *E. aerogenes*. Batch-cultivating experiments showed that by the overexpression of NadE (N), the hydrogen yields of IAM1183/N, IAM1183-CD/N, and IAM1183-CDE/N increased 1.06-, 1.35-, and 1.55-folds, respectively, compared with IAM1183. Particularly worth mentioning is that the strain IAM118-CDE/N reached 2.28 mol in H_2_ yield, per mole of glucose consumed. IAN1183/R, IAM1183-CD/R, and IAM1183-CDE/R showed increasing H_2_ yields in batch culture. Metabolic flux analysis indicated that increased expression of RyhB led to a significant shift in metabolic patterns. We further investigated IAM1183-CDE/N, which had the best hydrogen-producing traits, as a potential candidate for industry applications using a 5-L fermenter; hydrogen production reached up to 1.95 times greater than that measured for IAM1183.

**Conclusions:**

Knockout of *nuoCD* or *nuoCDE* and the overexpression of *nadE* in *E. aerogenes* resulted in a redistribution of metabolic flux and improved the hydrogen yield. Overexpression of RyhB had an significant change on the hydrogen production via NADH pathway. A combination of strategies would be a novel approach for developing a more economic and efficient bioprocess for hydrogen production in *E. aerogenes*. Finally, the latest CRISPR-Cas9 technology was successful for editing genes in *E. aerogenes* to develop our engineered strain for hydrogen production.

**Electronic supplementary material:**

The online version of this article (doi:10.1186/s13068-017-0938-2) contains supplementary material, which is available to authorized users.

## Background

The ongoing rapid increase in world energy demand is mostly accommodated by conventional fossil fuels, e.g., petroleum, natural gas, and coal. Indeed, fossil fuels account for approximately 80% of world energy consumption [1, 2]. However, fossil fuels are a finite and nonrenewable energy resource and have had a serious, negative influence on the environment owing to the consequent emission of greenhouse gases, e.g., CO_x_, NO_x_, and SO_x_. In response to this dual problem (i.e., depletion and pollution), scientists have become committed to exploring clean fuels that could substitute for fossil fuels [3, 4]. Among the potential clean fuels, hydrogen gas (H_2_), which produces only water when burned, is considered a clean, carbonless, and ideal alternative energy carrier because of its high conversion efficiency, recyclability, and environmentally friendly attributes [2, 3]. At present, however, hydrogen production is still highly dependent on fossil fuels. Therefore, new approaches for biohydrogen production, such as dark fermentation, photofermentation, and biophotolysis, have been widely investigated because the process of biohydrogen production could be environmentally sound [2, 5]. In particular, dark fermentative biohydrogen production from biomass also holds promise for developing new technologies suitable for industrial use [2, 5, 6].

*Enterobacter aerogenes* also called *Klebsiella aerogenes*, a facultative anaerobe, carries out fermentative hydrogen production similar to *Escherichia coli* and has a high H_2_ evolution rate; moreover, *E. aerogenes* grows rapidly during dark fermentation [6, 7]. This enterobacterium can produce H_2_ using a large range of carbon sources such as agriculture waste, industrial organic wastewater, or in combination with sewage [8–11], and *E. aerogenes* is regarded as an ideal strain for large-scale biohydrogen production. Therefore, it is necessary to delineate and understand the metabolic networks of hydrogen production from *E. aerogenes*.

**Fig. 1:**
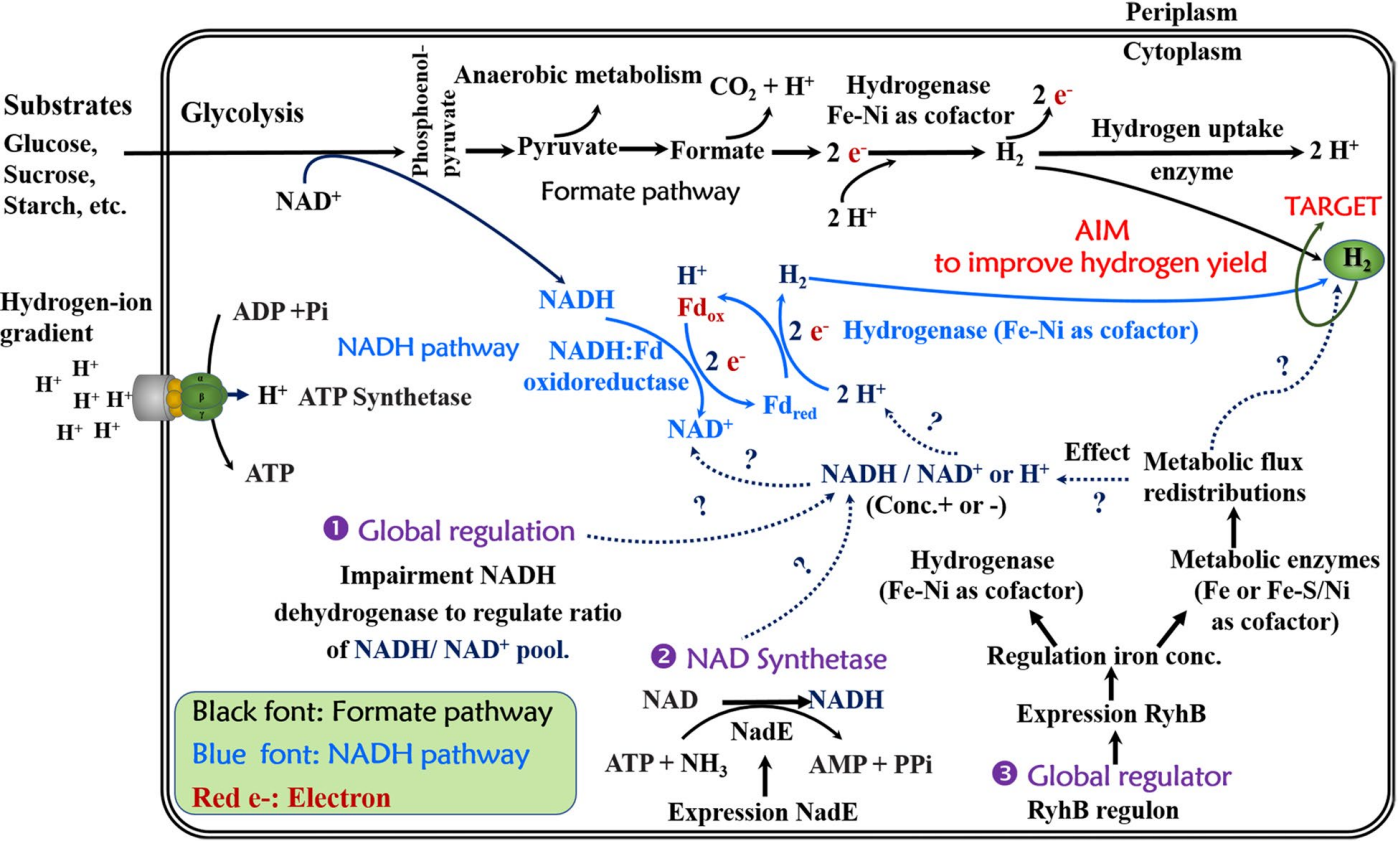
Diagram of primary anaerobic metabolic pathways for bacterial hydrogen production. Shown are three putative strategies to enhance hydrogen yield as outlined in this study. The diagram was adapted from references [8], [17], and [18]

Two metabolic pathways for producing hydrogen pertain to *E. aerogenes* [12, 13]. One is the formate pathway, which is based on the decomposition of formate mediated by the formate hydrogen lyase (FHL) system (Fig. 1) [13, 14]. The maximum yield of hydrogen by *E. aerogenes* through the formate pathway is 2 mol H_2_/mol glucose on account of the stoichiometry of the intracellular reactions. In our previous study, we knocked out the gene *hycA*, which encodes the formate hydrogenlyase repressor protein, and we also deleted the small subunit *hybO* of uptake hydrogenase [13]. It has been suggested that the hydrogen yield through the formate pathway can be enhanced by genetic manipulation of the FHL system. The other pathway is the NADH pathway (Fig. 1) [15], which can be regulated by altering the intracellular redox state, by external addition of NADH or NAD^+^, by blocking any pathway that would compete for NADH use, or by changing the NADH:NAD^+^ ratio during the production of hydrogen (Fig. 1) [12, 15, 16].

A substantial body of recent work has shown that small RNAs play an important role in regulating metabolism in bacteria and eukaryotes [17, 19]. RyhB, a small noncoding RNA, negatively regulates gene expression by base pairing with mRNAs to trigger their degradation via RNase E and RNase III [20]. RyhB is associated with various important cellular functions, including TCA cycle activity, resistance to oxidative stress, and iron homeostasis in many bacteria, such as *E. coli* and *Klebsiella pneumoniae* [17, 21, 22]. In *E. coli*, *hycA* and *hybO* are downregulated by RyhB under anaerobic conditions [23], suggesting that RyhB might influence the metabolic regulation of hydrogen production in *E. aerogenes*. Moreover, RyhB overexpression in *E. coli* conferred a sevenfold increase in succinate production, whereas no citrate was detected, and in acetate accumulated, while revealing the comprehensive effect of RyhB on glucose central metabolism [23]. Although the metabolic relevance of RyhB has been demonstrated in different species, the relationship between RyhB and hydrogen production capacity in *E. aerogenes* has not been studied.

Apart from the chemical reduction of various molecules to yield hydrogen molecules, the common metabolic cofactor NAD(H) is an indispensable participant in intracellular oxidation–reduction reactions in microbes [24, 25]. Cofactor engineering—an important branch of metabolic engineering—is accomplished mainly by changing the intracellular forms and levels of cofactors to manipulate metabolic fluxes for a particular metabolite or metabolic network [25–27]. In essence, the flow of electrons, H^+^, NADH-shuttled H^+^, or energy translates into the production of hydrogen or participates in hydrogen metabolism in anaerobic fermentation (Fig. 1). Therefore, it is also important to know how to regulate the intracellular redox state of NADH/NAD^+^ pools to promote hydrogen production.

In this study, we adopted three strategies (Fig. 1) to improve hydrogen production in the wild-type *E. aerogenes* strain IAM1183. The genes *nuoC*, *nuoD*, and *nuoE*, which belong to the NADH dehydrogenase cluster, were knocked out in IAM1183 via the CRISPR-Cas9 gene-editing approach [28, 29] to create the double mutant IAM1183-CD (*∆nuoC*/*∆nuoD*) and triple mutant IAM1183-CDE (*∆nuoC*/*∆nuoD*/*∆nuoE*). To regulate anaerobic metabolism by the small RNA RyhB [21, 23] in IAM1183 in order to increase hydrogen production, RyhB was overexpressed in each of IAM1183, IAM1183-CD, and IAM1183-CDE. Moreover, *nadE*, which encodes nicotinic acid phosphoribosyl transferase (NAD synthetase) [18, 26, 30] from *K. pneumonia*, was cloned and heterologously expressed in IAM1183 and its mutant strains to modulate the intracellular NAD(H)/NAD^+^ pool to promote hydrogen production during anaerobic fermentation. To quantify how RyhB or/and NadE regulate anaerobic metabolism in IAM1183 and its mutants, a metabolic network model was constructed to facilitate the calculation of changes in metabolic flux distributions in mutants. The activities of three key enzymes in NADH-consuming pathways were also measured to understand the redistribution of the intracellular reducing power in all mutants. Among the mutants, the strain that exhibited the greatest hydrogen production was determined to be a preferred candidate for industrial bioproduction of hydrogen and was further investigated with respect to its potential to generate hydrogen in a fermenter. These results reveal the effect of *nuoCDE*, RyhB, and NadE on hydrogen production and how the modulation of intracellular NADH concentration influences the H_2_ yield from *E. aerogenes*.

## Methods

### Strains, plasmids, and materials

Table 1 lists the bacterial strains and plasmids used in this study. *E. aerogenes* IAM1183 was received as gift from the Green Industry Biotechnology Lab of Tsinghua University. *E. coli* DH5α and *E. coli* JM109 as host strains for genetic manipulation were purchased from TaKaRa Biotechnology (Dalian, China). Kits for genomic DNA purification, plasmid DNA purification, and DNA gel extraction were purchased from GenScript (USA). The Gibson assembly master mix kit, all restriction enzymes, DNA polymerase, and DNA-modifying enzymes were obtained from New England Biolabs (Beverly, MA, USA). Yeast extract and tryptone were acquired from Thermo Fisher Biochemicals (Beijing). Unless specifically indicated otherwise, all other chemicals were obtained from Sigma-Aldrich (St. Louis, MO, USA).

**Table 1:** Strains and plasmids used in this study

Strain or plasmid	Genotype and relevant characteristics	Source or literature
Strains		
IAM1183	Wild type	IAM (Tokyo, Japan)
IAM1183/N	Carrying plasmid pET-28a-nadE	This study
IAM1183/R	Carrying plasmid pKK102-ryhB-cm	This study
IAM1183/NR	Carrying plasmid pET-28a-nadE and pKK102-ryhB-cm	This study
IAM1183-CD	∆*nuoC*/∆*nuoD*	This study
IAM1183-CD/N	∆*nuoC*/∆*nuoD*, carrying plasmid pET-28a-nadE	This study
IAM1183-CD/R	∆*nuoC*/∆*nuoD*, carrying plasmid pKK102-ryhB-cm	This study
IAM1183-CD/RN	∆*nuoC*/∆*nuoD*, carrying plasmid pET-28a-nadE and pKK102-ryhB-cm	This study
IAM1183-CDE	∆*nuoC*/∆*nuoD*/∆*nuoE*	This study
IAM1183-CDE/N	∆*nuoC*/∆*nuoD*/∆*nuoE*, carrying plasmid pET-28a-nadE	This study
IAM1183-CDE/R	∆*nuoC*/∆*nuoD*/∆*nuoE*, carrying plasmid pKK102-ryhB-cm	This study
IAM1183-CD/RN	∆*nuoC*/∆*nuoD*/∆*nuo*E, carrying plasmid pET-28a-nadE and pKK102-ryhB-cm	This study
*E. coli* DH5α	*F*-, *φ 80dlacZ ΔM15*, Δ (*lacZYA* -*argF*) *U169*, *deoR*, *recA1*, *endA1*, *hsdR17* (*rK*-, *mK* +), *phoA*, *supE44*, *λ*-, *thi* -*1*, *gyrA96*, *relA1*	TaRaKa
*E. coli* JM109	*recA1*, *endA1*, *gyrA96*, *thi*-*1*, *hsdR17* (*rk*-*mk* +), *e14*-(*mcrA*-) *supE44*, *relA1*, Δ(*lac*-*proAB*)*/F’*[*traD36*, *proAB*+, *lacIq*, *lacZ ΔM15*]	TaRaKa
*K. pneumoniae* nov. sp	Wild type	This study
Plasmid		
pCAS9	*repA101*(*Ts*) *kan Pcas*-*cas9 ParaB*-*Red lacIq Ptrc*-*sgRNA*-*pMB1*	[28]
PTargetF-cat	pMB1 aadA sgRNA-cat	[28]
PTargetF-nuoN20	pMB1 aadA sgRNA-nuoN20	This study
pET-28a	N-His, N-Thrombin, N-T7, C-His, Kan^r^	TaRaKa
pSTV28	*Cm* ^*r*^, with multiple coning sites	TaRaKa
pMD18-T Vector	TA Cloning vector, *Amp* ^*r*^	TaRaKa
pMD18-T-nadE	*Amp* ^*r*^, derived from pMD18-T inserted *nadE*	This study
pET-28a-nadE	*nadE*, *Kan* ^*r*^ derived from pET-28a	This study
pKK102-ryhB	RyhB, *Amp* ^*r*^	[23]
pKK102-ryhB-cm	RyhB, *Cm* ^*r*^ derived pKK102-ryhB	This study

### Culture medium and cultivation

Luria–Bertani broth containing 10 g L^−1^ tryptone, 5 g L^−1^ yeast extract, and 10 g L^−1^ NaCl was used to culture cells for genetic engineering and culture maintenance. *Enterobacter aerogenes* IAM1183, its mutants, and *E. coli* as host for genetic manipulation were grown in Luria–Bertani medium at 37 °C or 30 °C, supplemented with ampicillin (100 µg mL^−1^), kanamycin (25 or 50 µg mL^−1^), spectinomycin (50 µg mL^−1^), or chloramphenicol (25 µg mL^−1^), as required. Glucose medium containing 15 g L^−1^ glucose, 5 g L^−1^ tryptone, 14 g L^−1^ K_2_HPO_4_, 6 g L^−1^ KH_2_PO_4_, 2 g L^−1^ (NH_4_)_2_SO_4_, and 0.2 g L^−1^ MgSO_4_·7H_2_O 0 was used for hydrogen production, metabolic flux analysis, and bioreactor experiments.

### Genetic manipulation

Table 2 lists detailed information for the primers used. Standard molecular genetics techniques were employed [32]. Genomic DNA from *E. aerogene*s IAM1183 was prepared as described [32] or per the kit manual supplied by the manufacturer. Plasmid DNA was purified using the QIAprep Spin Miniprep kit. The DNA was extracted from gel pieces using the GenScript DNA gel extraction kit. Amplification was carried out using a PCR apparatus from Bio-Rad (Hercules, CA, USA) with a final volume of 50 µL containing 5 µL of 10× PCR reaction buffer, 10 mM of each dNTP, 30–60 ng DNA template, 10 pmol each of the appropriate primers, and 0.5 U each of Taq polymerase and Pfu DNA polymerase (New England Biolabs). DNA sequencing was performed by Life Technologies (Shanghai Invitrogen, China).

**Table 2:** Primers used in the experiments

Primers	Oligonucleotide sequence (from 5′ to 3′)	PCR fragment	Source
F-CDuh	CCCTGCAACTTCGGCCTGTCA	Upstream-homologous arm of *nuoB*	This study
R-CDuh	TGATTCTCGTGCATTTAAATCTCGTCCGGTGT		This study
F-CDdh	ACCGGACGAGATTTAAATGCACGAGAATCAAC	Downstream-homologous arm of *nuoE*	This study
R-CDdh	CTGCTCCAACAGGTCAGGAATCG		This study
F-CDEuh	AGCAAGAAGTCAATAAAAGCGT	Upstream-homologous arm of *nuoB*	This study
R-CDEuh	ATCACTGTCTTCATTTAAATCTCGTCCGGTGT		This study
F-CDEdh	CGAGATTTAAATGAAGACAGTGATCCGCA	Downstream-homologous arm of *nuoF*	This study
R-CDEdh	ACGGCCTTCAAGGGAGTTAATC		This study
F-nuoCD	TCCTAGGTATAATACTAGTGGCGAACTGCGCAATCGTTTTGGGTTTTAGAGCTAGAAATAGC	nuoCD-N20	This study
R-nuoCD	ACTAGTATTATACCTAGGACTGAGCTAGCTGTCAAG		This study
F-nadE	CCG*GAATTC*ATGACGCTACAACAAGAGATT	*nadE* ORF fragment	This study
R-nadE	CCC*AAGCTT*CTATTTTTTCCAGAAATCGTC		This study
F-p102-Chol	TGCACG*GTGCAC*AGTGCCAAGC TTGCATGCCT	*Chol* ORF fragment	This study
R-p102-Chol	CGGCAT*GACTCCCCGTC*AGTATACACTCCGCTAGCGC		This study

Relevant restriction enzyme sites are underlined and in italics

**Fig. 2:**
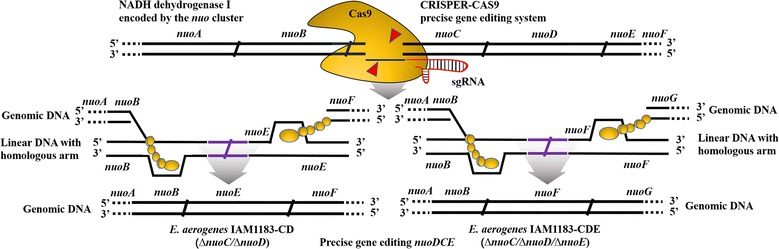
Schematic diagram showing the ∆nuoCD and ∆nuoCDE mutants constructed using the CRISPR-Cas9 method

Based on the CRISPR-Cas9 gene-editing procedure [9, 28], the linear pTargetF-cat carrying nuoN20 was amplified from pTargetF-cat using primers F-nuoCD and R-nuoCD, then the linear pTargetF-cat carrying nuoN20 was self-assembled using the Gibson assembly method [31], yielding plasmid pTargetF-nuoN20. The linear upstream-homologous arm (*nuoB*) fragment and downstream-homologous arm (*nuoE*) fragment were amplified from the IAM1183 genome using the primers F-CDuh and R-CDuh as well as F-CDdh and R-CDdh. The donor linear DNA segment was constructed using the obtained purified products of the *nuoB* fragment and *nuoE* fragment by overlap extension using PCR. Plasmid pCas9 was electroporated into IAM1183 competent cells using the Bio-Rad Micro-Pulser Electroporator at 1800 V, and l-arabinose (10 mM final concentration) was added to the culture to induce λ-Red recombinase expression. For electroporation, 50 µL IAM1183 competent cells harboring pCas9 was mixed with 100 ng pTargetF-nuoN20 plasmid DNA and 400 ng donor DNA in a 2-mm Gene Pulser cuvette (Bio-Rad). The positive mutants were preliminarily screened by colony PCR. The standard method of *nuoC/D/E* gene knockout was the same as the above protocol, and the same pTargetF-nuoN20 was used to knock out *nuoC/D/E*. The linear upstream-homologous arm (*nuoB*) and downstream-homologous arm (*nuoF*) were amplified from the IAM1183 genome using primers F-CDEuh and R-CDEuh as well as F-CDEdh and R-CDEdh. Figures 1 and 2 illustrate the process by which *nuoC/D/E* loci were deleted. IAM1183 mutants with a disrupted *nuoCD* or *nuoCDE*, named IAM1183-CD or IAM1183-CDE, respectively, were further verified via sequencing by Life Technologies.

### Overexpressions of *ryhB* and NadE

The plasmid pKK102-ryhB-cm was derived from plasmid pKK102-ryhB with ampicillin resistance to overexpress RyhB in *E. aerogenes*. First, the chloramphenicol cassette was amplified from pSTV28 plasmid DNA using primers F-p102-Chol and R-p102-Chol. The PCR fragment was double digested using restriction enzymes, *spA*lI and *Hin*dIII. The purified fragments containing the chloramphenicol cassette were inserted into pKK102-ryhB that had been digested with *spA*lI and *Hin*dIII to replace the ampicillin cassette to create pKK102-ryhB-cm. Plasmid pKK102-ryhB-cm was first transformed into *E. coli* DH5α or *E. coli* JM109, and positive colonies were screened on Luria–Bertani agar plate containing chloramphenicol (25 µg mL^−1^). Then, the purified pKK102-ryhB-cm was electrotransformed into IAM1183, IAM1183-CD, and IAM1183-CDE to generate IAM1183/R, IAM1183-CD/R, and IAM1183-CDE/R, respectively. When necessary, 0.6 g L^−1^ l-arabinose was added to induce RyhB expression.The RyhB expression in IAM1183/R was analyzed by northern blot (see Additional file 1: Figure S2).

Plasmid pET-28a-nadE, which was used to heterologously express NadE in *E. aerogenes*, was constructed via the following steps. The *nadE* open reading frame was amplified from the chromosome of *K. pneumoniae* nov. sp using primers F-nadE and R-nadE. The PCR product was inserted into pMD18-T and further confirmed by sequencing. The *Eco*RI and *Hin*dIII double-digested restriction fragments from pMD18-T-nadE were harvested and inserted into pET-28a that had been digested with the same restriction enzymes to create the expression plasmid pET-28a-nadE, which was first transformed into *E. coli* DH5α or *E. coli* JM109 for cultivation with 25 mg L^−1^ kanamycin. Then, the purified pET-28a-nadE plasmid DNA was electroporated into IAM1183, IAM1183-CD, and IAM1183-CDE to generate IAM1183/N, IAM1183-CD/N, and IAM1183-CDE/N, respectively. NadE overexpression was induced by the addition of 0.1 mM (final concentration) isopropylthio-galactoside.The NadE expression in IAM1183/N was checked by SDS-PAGE (see Additional file 1: Figure S1).

### Assays for enzyme activities

All enzyme activities were measured under anaerobic conditions, and the reaction systems were prepared in an anaerobic chamber (Hengyue YQX- I, China) [33]. Bacterial cells were harvested by centrifugation for 10 min at 12,000 rpm at 4 °C after anaerobic cultivation for 8 h, and the pellet was resuspended in 100 mM potassium phosphate (pH 7.0). Cell-free extracts were prepared by ultrasonication (Ultrasonicator, FS30H, Fischer Scientific, Pittsburgh, PA) on ice. This approach allows enzyme activities to be measured by monitoring the decrease in absorbance of NADH (*ε*_340_ = 6.3 mM^−1^ cm^−1^) via a spectrophotometer (Antpedia, UV-721G, China). Lactate dehydrogenase (LDH; EC 1.1.1.27) activity was determined by measuring the decrease in NADH absorbance via a reaction system containing 1 mL imidazole–HCl (pH 6.7), 50 μL of 10 mM NADH, 1.5 mL of 0.5 mM sodium pyruvate, and 0.5 mL cell-free extract. Alcohol dehydrogenase (ADH; EC 1.1.1.1) activity and 2,3-butanediol dehydrogenase (BDDH; EC 1.1.1.76) activity were measured in a manner similar to that of LDH, except that 1.5 mL of 0.5 mM sodium pyruvate was substituted with 1.5 mL of 0.5 mM acetaldehyde and 1.5 mL of 10 mM acetoin, respectively.

### Anaerobic serum-bottle batch cultivation for hydrogen production

All strains were cultured using 100-mL serum bottles with top caps and PTFE/silicone rubber septa in an anaerobic environment. Each bottle included 30 mL of fermentation medium. The air in the bottle and the small amount of oxygen in the fermentation medium were purged with aseptic nitrogen gas for 10 min to ensure anaerobic growth. The serum bottles were cultivated at 37 °C and 200 rpm for 20 h. The gas produced after 20 h of fermentation was passed through 5 M sodium hydroxide (1000-mL blue reagent bottle containing 500 mL 5 M sodium hydroxide solution) to clear any carbon dioxide.

### Fermentation for hydrogen production

Fermentation experiments were performed at 37 °C with agitation at 250 rpm in a 5-L fermenter (Biostat A, Sartorius, Germany) containing 3 L medium without a pH control. Aseptic nitrogen was sparged for 90 min to purge residual air from the fermenter to ensure an anaerobic environment. The fermenter was then inoculated with an overnight seed broth at an inoculum size of 1.7% (v/v) and cultivated at 37 °C for 40 h. Carbon dioxide was absorbed via 5 M sodium hydroxide, and hydrogen production was measured using a wet-gas meter (Colom BSD0.5, China). Cell density and pH were monitored continuously. Samples were taken every 2 h. Concentrations of metabolites were determined using a high-performance liquid chromatography (HPLC) system (Shimadzu 10A, Japan).

### Analysis of H_2_ and metabolites

Cell density was measured at 600 nm (OD_600_) with the UV-721G spectrophotometer. Dry cell weight was subsequently calculated as: dry cell weight (mg mL^−1^) = 0.132 × OD_600_ for *E. aerogenes* [8]. The total volume of gas produced in the anaerobic serum bottle was measured via displacing pure water within an inverted cylinder, and the total volume of hydrogen produced via the 5-L fermenter was measured using a wet-gas meter.

Components of the gas included ethanol and 2,3-butanediol, which were quantified using a gas chromatographic apparaus (Shimadzu GC-2010 Plus) equipped with a Parapak Q column and a thermal conductivity detector, with He as the carrier gas. The working temperature of the detector was 40 °C and that of the column was 200 °C.

A 10 mL sample drawn from the chemostat cultures was centrifuged at 12,000 rpm for 15 min at 4 °C, and the supernatant was collected for analysis of residual glucose and metabolites. The concentration of each of acetate, formate, lactate, citric acid, acetoin, succinate, and alcohols in the culture medium was determined using an HPLC system (Shimadzu LC-20A). The system was equipped with a C-18 column (Shimadzu PREP-ODS(H) KIT) and a refractive index detector (Shimadzu RID-10A). A 0.2% aqueous phosphoric acid solution was used as the mobile phase. The column temperature was 33 °C, and the flow rate was 0.8 mL min^−1^. The retention times for succinate, lactate, formate, acetate, 2,3-butanediol, and ethanol were 9.747, 3.884, 4.350, 5.760, 6.426, 8.346, and 3.290 min, respectively.

## Results and discussion

### Disruption of nuoCDE with the CRISPR-Cas9 gene-editing system

The CRISPR-Cas9-based precise gene-editing system [28, 29] was employed to knockout *nuoC, nuoD,* and *nuoE* (the NADH dehydrogenase subunits in IAM1183) to systematically examine the effect of NADH dehydrogenase level on hydrogen production and carbon flux redistribution under anaerobic conditions (Figures 1 and 2). The standard gene-editing protocols for disruption of the *nuoCDE* genes are summarized in Fig. 2 [21]. Positive mutants were identified by colony PCR using primers F-CDuh/R-CDdh or F-CDEuh/R-CDEdh. Approximately 1–2% of colonies had a positive signal, implying that one or two colonies were presumably correct. The positive colonies were sent to Sangon Biotech for sequencing, and the results suggested that *nuoCD* and *nuoCDE* were indeed knocked out of the IAM1183 genome, and that the CRISPR-Cas9 system could be used as a powerful genetic manipulation tool to precisely edit genes in *E. aerogenes*. The positive transformants in which *nuoCD* and *nuoCDE* were deleted were named IAM1183-CD and IAM1183-CDE, respectively.

### Characterization of all mutants and measurement of hydrogen production

**Fig. 3:**
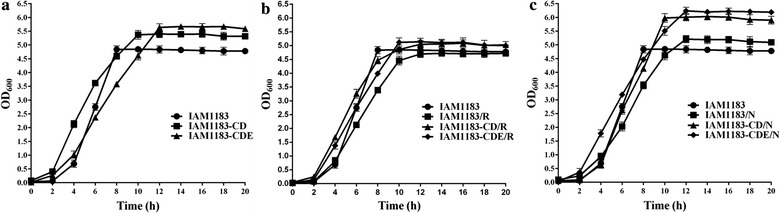
Comparison of cell growth (in terms of OD600) for IAM1183 and its mutants. **a** Growth curves for IAM1183-CD and IAM1183-CDE. **b** Growth curves for IAM1183/R, IAM1183-CD/R, and IAM1183CDE/R. **c** Growth curves for IAM1183/N, IAM1183-CD/N, and IAM1183-CDE/N

All strains were cultivated to measure hydrogen production in 100-mL serum bottles with 30 mL glucose medium under anaerobic conditions [13]. The wild-type IAM1183 served as the control. According to the growth curves for all strains (Fig. 3), after 24 h of culture, the cell density was greater for both strains ∆*nuoCD* and *∆nuoCDE* compared with IAM1183 (Fig. 3a). The exponential phase of each of IAM1183-CD and IAM1183-CDE was estimated by recording the time when the cell density reached 95 and 75% of maximum, respectively (Fig. 3a). Compared with the 8-h exponential phase of IAM1183, the exponential phases of mutants IAM1183-CD and IAM1183-CDE were prolonged by ~ 2 and ~ 4 h, respectively (Fig. 3a). The density of each of IAM1183-CD/R and IAM1183-CDE/R was somewhat greater than the respective IAM1183 and IAM1183/R at the end of the 24-h cultivation. The final OD_600_ value for IAM1183 did not differ from that of IAM1183/R (Fig. 3b). Based on OD_600_, the final densities of IAM1183/N, IAM1183-CD/N, and IAM1183-CDE/N were greater than that of IAM1183 by 1.11-, 1.26-, and 1.40-fold, respectively. Moreover, compared with the 8-h plateau phase of IAM1183, the plateau phase of the three mutants was delayed by ~ 2–4 h (Fig. 3c). These results demonstrated that expression *nadE* in IAM1183 and in *∆nuoCD* or *∆nuoCDE* had a positive effect on fermentation. The trends of growth characteristics of all ∆*nuoCD* and *∆nuoCDE* mutants were similar (Fig. 3), implying that RyhB level did not affect cell growth in these strains.

**Fig. 4:**
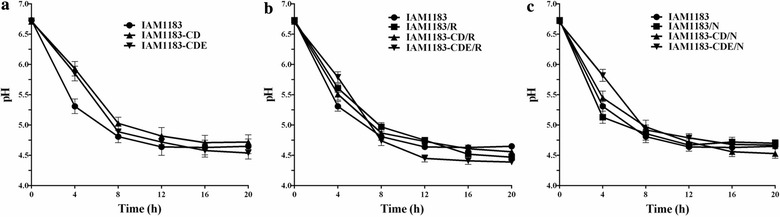
Comparison of pH of the medium over time for IAM1183 and its mutants during the 20-h batch cultivation. **a** The pH curves for IAM1183CD and IAM1183CD. **b** The pH curves for IAM1183/R, IAM1183-CD/R, and IAM1183-CDE/R. **c** The pH curves for IAM1183/N, IAM1183-CD/N, and IAM1183-CDE/N

During the 20-h batch cultivation, the pH of the culture medium of each mutant except IAM1183/N decreased at a slower rate compared with IAM1183 (Fig. 4). However, the final pH of the medium differed by only ~ 0.33 among the strains (Fig. 4). Comparison of pH for IAM1183 and its mutants, and the pH values of accurate cultivating time at 4-h and final 20-h are summarized as shown in Table 3. The pH values for the mutants (range, 5.45 ± 0.07 to 5.93 ± 0.04) except for IAM1183/N (5.13 ± 0.07) during exponential growth (0–8 h) were higher than for IAM1183 (5.31 ± 0.12). These results were particularly relevant for potential application of these mutants in large-scale H_2_ production (Fig. 4 and Table 3) [13, 15]. This apparent elevation in the medium pH for the mutants might be one of the reasons why the final OD_600_ values for all mutants were somewhat higher than that for strain IAM1183 (Fig. 3). After 20 h of cultivation, the final values of pH (range, 4.39 ± 0.09 to 4.72 ± 0.09) for all three mutants were close to that of IAM1183 (4.65 ± 0.08), i.e., within the margin of xperimental error (Fig. 4 and Table 3). These observations suggest that disruption of NADH metabolism did not have a negative effect on the pH of medium.

**Table 3:** Comparison of the optimal pH for growth between IAM1183 and its mutants at the midpoint of exponential growth and after 20 h of batch cultivation

	Strain								
	IAM1183	IAM1183/N	IAM1183/R	IAM1183-CD	IAM1183-CD/R	IAM1183-CD/N	IAM1183-CDE	IAM1183-CDE/R	IAM1183-CDE/N
Exponential phase (4-h)	5.31 ± 0.12	5.13 ± 0.07	5.61 ± 0.1	5.93 ± 0.04	5.51 ± 0.16	5.45 ± 0.07	5.85 ± 0.19	5.79 ± 0.11	5.82 ± 0.15
Stationary phase (20-h)	4.65 ± 0.08	4.7 ± 0.06	4.47 ± 0.14	4.72 ± 0.09	4.56 ± 0.13	4.53 ± 0.12	4.54 ± 0.17	4.39 ± 0.09	4.67 ± 0.08

**Fig. 5:**
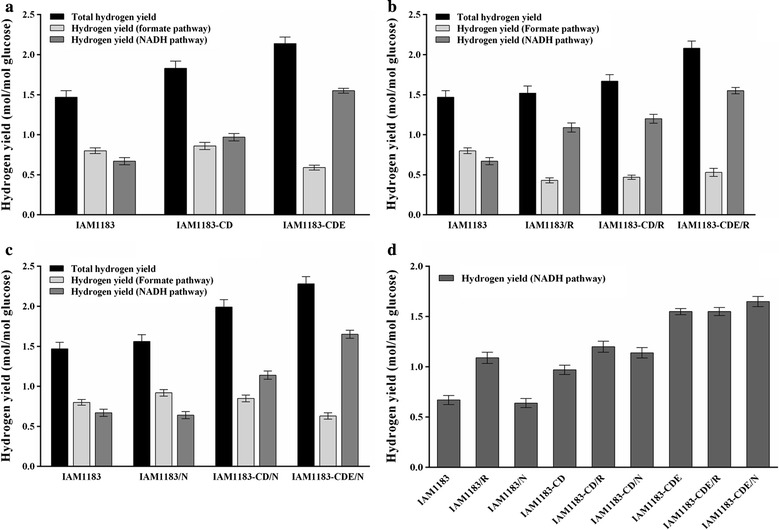
The hydrogen production from glucose compared by IAM1183 among all strains in serum bottle batch assays (*n* = 3). The yield of H_2_ generated through the formate pathway can be calculated by the following equation *V*_*h*_ = *V*_*e*_ + *V*_*a*_ − *V*_*f*_ , where *V*_*h*_ is the amount of hydrogen produced through the formate pathway, *V*_*e*_ is the amount of ethanol, *V*_*a*_ is the amount of acetate, and *V*_*f*_ is the amount of formate. **a** Hydrogen evolved from IAM1183-CD and IAM1183-CDE. **b** Hydrogen evolved from IAM1183/R, IAM1183-CD/R, and IAM1183-CDE/R. **c** Hydrogen evolved from IAM1183/N, IAM1183-CD/N, and IAM1183-CDE/N. **d** Special illustration of hydrogen evolved via the NADH pathway

As shown in Fig. 5, each mutant produced significantly more hydrogen than IAM1183. The hydrogen production of per mole of glucose consumed by mutants IAM1183-CD and IAM1183-CDE was 26.2 and 47.6% greater compared with the wild-type stain (Fig. 5a and Table 4). Moreover, for IAM1183-CDE compared with IAM1183, hydrogen produced via the NADH pathway was significantly elevated (2.31-fold, Fig. 5d) and that via the formate pathway significantly reduced (1.36-fold, Fig. 5a). A previous study reported that *nuoE* encodes the NADH dehydrogenase module bearing the NADH-binding and FMN-binding sites and four Fe-S clusters [34], implying that *nuoE* may play major role in the NADH pathway in *E. aerogenes.* These results suggest that components encoded by *nouC*, *nouD*, and *nouE* have an apparent effect on hydrogen production in *E. aerogenes*.

**Table 4:** Analysis of consumed substrate and the production of anaerobic metabolites after 20 h of cultivation in a serum bottle for wild-type IAM1183 and mutants (*n* = 3)

End product	Strain								
	IAM1183	IAM1183/R	IAM1183/N	IAM1183-CD	IAM1183-CD/R	IAM1183-CD/N	IAM1183-CDE	IAM1183-CDE/R	IAM1183-CDE/N
Glucose consumed (mM)	57.42 ± 1.38	59.27 ± 0.69	60.62 ± 0.85	70.02 ± 0.66	68.13 ± 0.94	74.05 ± 0.43	69.02 ± 0.77	69.28 ± 0.49	71.09 ± 0.39
Residual sugar (mM)	4.66 ± 0.25	4.33 ± 0.12	4.09 ± 0.15	2,4 ± 0.12	2.74 ± 0.17	1.67 ± 0.08	2.58 ± 0.14	2.53 ± 0.09	2.2 ± 0.07
2,3-Butanediol (mM)	9.35 ± 1.56	11.86 ± 0.91	11.53 ± 0.78	11.73 ± 1.37	24.75 ± 0.42	13.79 ± 1.09	8.26 ± 0.17	11.96 ± 0.78	10.69 ± 0.26
Lactate (mM)	23.62 ± 0.46	44.15 ± 0.99	31.54 ± 0.96	18.78 ± 1.03	25.33 ± 0.81	22.31 ± 0.66	32.14 ± 0.29	36.61 ± 0.45	38.21 ± 0.36
Acetate (mM)	17.27 ± 0.32	10.36 ± 0.39	20.31 ± 0.65	27.96 ± 0.69	23.17 ± 0.55	30.52 ± 1.26	17.16 ± 0.31	15.27 ± 0.13	16.01 ± 0.63
Formate (mM)	2.24 ± 0.17	3.92 ± 0.77	2.83 ± 0.98	6.19 ± 0.76	5.98 ± 0.47	8.28 ± 1.62	3.67 ± 0.34	2.92 ± 0.19	1.63 ± 0.27
Succinate	1.86 ± 0.08	1.01 ± 0.11	2.31 ± 0.19	3.22 ± 0.17	3.02 ± 0.12	3.59 ± 0.36	3.79 ± 0.48	3.02 ± 0.12	3.86 ± 0.23
Ethanol (mM)	31.93 ± 1.2	13.85 ± 2.02	38.09 ± 0.98	38.69 ± 1.16	14.47 ± 1.32	40.87 ± 0.72	25.14 ± 0.26	23.49 ± 1.19	29.96 ± 0.77
Citric acid (mM)	1.68 ± 0.25	1.52 ± 0.49	1.43 ± 0.33	1.69 ± 0.37	1.51 ± 0.72	1.65 ± 0.96	1.57 ± 0.67	1.62 ± 0.22	1.64 ± 0.93
Acetoin (mM)	25.71 ± 1.11	19.21 ± 0.58	27.32 ± 0.64	20.99 ± 0.84	20.13 ± 0.74	25.02 ± 0.17	23.19 ± 0.29	26.81 ± 0.44	25.62 ± 0.51
H_2_ yield (mol/mol glucose)	1.47 ± 0.06	1.52 ± 0.13	1.56 ± 0.17	1.83 ± 0.09	1.67 ± 0.41	1.99 ± 0.22	2.14 ± 0.69	2.08 ± 0.12	2.28 ± 0.12

However, overexpression of RyhB in IAM1183/R caused only a modest increase in hydrogen production (3.7%) compared with IAM1183. For IAM1183/R, hydrogen produced via the formate pathway decreased by 60% compared with IAM1183 (Fig. 5b), implying that RyhB represses the formate pathway in *E. aerogenes*. As the previous study reported that the expression of *hycA* gene, the operon of formate lyase, was decreasing [17], which maybe explain the above result. Besides, in comparison with IAM1183-CD, the production of H_2_ decreased by 7.2% of IAM1183-CD/R, and the strain IAM1183-CDE/R had a slight decrease on H_2_ production compared with IAM1183-CDE (Table 3). RyhB suppresses the expression of at least 44 genes under anaerobic conditions, including Fe-binding proteins and factors involved in the TCA cycle, and this fact may explain these differences in H_2_ production [17]. These unusual results may be due to a blockade of the ethanol biosynthesis pathway under anaerobic conditions, in which NADH is consumed more rapidly compared with other NADH-consuming pathways.

Cofactor engineering is accomplished mainly by changing the intracellular forms and levels of cofactors to manipulate metabolic fluxes for a particular metabolite or metabolic network [25, 26]. Expression of NadE regulates the NADH/NAD^+^ pool and reconstitutes the metabolic flux to promote hydrogen production. In our present study, compared with IAM1183, overexpression of NAD synthetase led to a significant increase in hydrogen yields for strains IAM1183/N (+ 6%), IAM1183-CD/N (+ 35%), and IAM1183-CDE/N (+ 55%) (Table 4 and Fig. 5c). Moreover, 15% more hydrogen was produced by the formate pathway in IAM1183/N compared with IAM1183 (Fig. 5c). These results did correspond with previous reports, in which supplementation of the culture medium with NADH led to a decrease in hydrogen production via the NADH pathway and an increase in production via the formate pathway [13, 15, 16]. Interestingly, however,, 17.5% more hydrogen was produced by the NADH pathway in IAM1183-CD/N compared with IAM1183-CD. The result could be due to the impairment of NADH dehydrogenase by knockout *nuoC/D*, which could influence the intracellular redox state of NADH. We also found that hydrogen production from IAM1183-CDE/N was 1.07-fold greater compared with IAM1183-CDE and that the concentrations of acetate and formate were 6.7 and 55.6% lower, respectively (Table 4), indicating that NADH provided via supplementation not only is used by the NADH pathway to produce hydrogen but also affects the intracellular metabolism of *E. aerogenes*. The results of these experiments demonstrate that hydrogen production can be enhanced by increasing the total intracellular pool of NAD(H).

It was very meaningful for evaluating the potential to regulate NADH for biohydrogen production. The hydrogen produced via the NADH pathway of all strains is compared in Fig. 5d. For the NADH pathway, IAM1183-CD and IAM1183-CDE produced 1.45-fold and 2.31-fold more hydrogen than IAM1183 (Fig. 5d). Apparently, the *nuoCDE* knockout substantially increased the conversion of NADH to H_2_ via the NADH pathway, implying that *nuoE* normally downregulates the hydrogen production via the NADH pathway in *E. aerogenes*. Moreover, the hydrogen produced via the NADH pathway of IAM1183/R, IAM1183-CD/R, and IAM1183-CDE/R was also increased by 1.63-, 1.23-, and 1.01-fold compared with IAM1183, IAM1183-CD, and IAM1183-CDE, respectively (Fig. 5d), which demonstrates that RyhB can enhance hydrogen production via the NADH pathway. However, the NadE expressed in IAM1183 and its mutants seemed to play a lesser role in converting NADH to H_2_ via the NADH pathway. The hydrogen produced via the NADH pathway of IAM1183/N was 4.5% lower compared with IAM1183, and for strains IAM1183-CD/N and IAM1183-CDE/N there was no significant difference in H_2_ yield via the NADH pathway (Fig. 5d), which is consistent with previous results [35]. These results suggest that disruption of *nuoCDE* could be an effective approach for promoting the conversion of NADH to hydrogen and overexpression of RyhB had a profound influence on the way of formation in hydrogen production.

### Analysis of the metabolite flux

Table 4 reports the final concentrations of the major excreted metabolites of all strains. The yields of 2,3-butanediol, acetate, and ethanol for IAM1183-CD were greater than for IAM1183, whereas the yield of lactate was lower, indicating that the lactate pathway was repressed by the double knockout of *nuoC* and *nuoD*. Moreover, IAM1183-CDE had significantly elevated concentrations of lactate (36%) and formate (64%) compared with IAM1183, whereas concentration of each of acetate (1%) and 2,3-butanediol (12%) was significantly lower. The fact that all these metabolites are produced through NADH-consuming pathways implies that *△nuoCD* and *△nuoCDE* mutants had altered intracellular NADH/NAD^+^ pools and underwent a redistribution of metabolic fluxes accordingly. Meanwhile, the relatively rapid growth of IAM1183-CD and IAM1183-CDE cells compared with IAM1183 indicated that the exponential growth phase was prolonged in these two mutants (Fig. 3a), which was presumably due to the decrease in the level of NADH dehydrogenase in these two mutants and the consequent reduction in energy conservation during exponential growth.

**Fig. 6:**
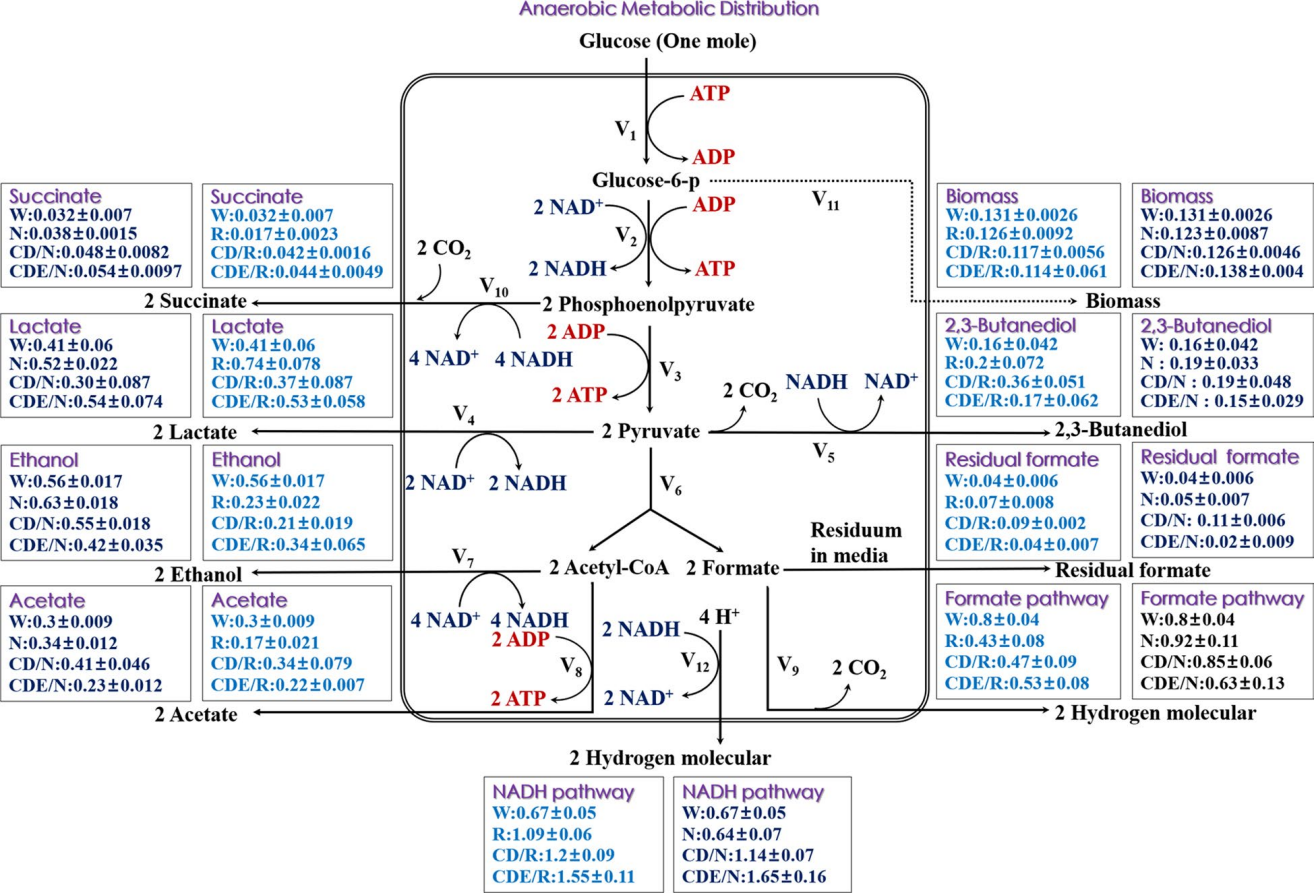
Metabolic distribution of IAM1183 upon overexpression of the small RNA RyhB and NadE after 20 h of cultivation (*n* = 3). The unit is mole. Redox state balance was calculated as the sum of the products with positive oxidation states and those with negative oxidation states per mole of consumed glucose (W IAM1183, R IAM1183-R, CD/R IAM1183-CD/R, IAM1183-CDE/R, N IAM1183-N, CD/N IAM1183-CD/N, and IAM1183-CDE/N)

RyhB has a global cellular effect on glucose metabolism [23]. We thus analyzed the metabolic flux of IAM1183/R, IAM1183 CD/R, and IAM1183-CDE/R. Compared with IAM1183, IAM1183/R yielded a greater amount of each of 2,3-butanediol (+ 26%) and lactate (+ 86%) (Table 4), whereas the concentrations of other metabolic products were lower compared with IAM1183. These results suggest that RyhB alters the size of the intracellular pool of NADH or NAD^+^, thereby affecting H_2_ productivity. Compared with IAM1183, IAM1183-CD/R produced significantly greater concentrations of acetate (+ 34.2%), lactate (+ 7.2%), 2,3-butanediol (+ 165%), and formate (+167%) yet produced less ethanol (− 55%) (Table 4). A previous study suggested that NAD^+^ regeneration tended to be quicker in the presence of excess reducing equivalents [35], and the results of our experiments showed that IAM1183-CD/R had a significantly altered composition of the intracellular NADH/NAD^+^ pools when the concentration of NADH increased above the physiologically normal level. We also compared the metabolic landscape of strain IAM1183-CD/R with that of IAM1183-CD, noting that ethanol production in both strains was ~ 63% lower compared with the wild-type strain IAM1183 and that ADH activity in IAM1183 was 1.24, 1.1, and 1.03 times that measured in IAM1183/R, IAM1183-CD/R, and IAM1183-CDE/R, respectively. Moreover, the ethanol concentration of mutant IAM1183-CDE/R was 6.6% lower than that of IAM1183-CDE. These results indicate that RyhB represses ethanol biosynthesis. The anaerobic metabolic flux of one mole of glucose, as affected by RyhB, was further analyzed to reveal the effect on IAM1183. As shown in Fig. 6, overexpression of *ryhB* altered the synthesis of all metabolites in IAM1183. This demonstrates that *ryhB* could alter the expression of genes that control the redox state of anaerobic metabolism and thereby downregulate the production of the terminal electron acceptor, i.e., H^+^, for hydrogen evolution under anaerobic conditions.

Overexpression of NAD synthetase led to a significant change in the concentrations of fermentation-related metabolites in strain IAM1183-CD/N. Compared with IAM1183/CD, there were increases in 2,3-butanediol (17.6%), acetate (9.2%), ethanol (5.6%), formate (33.8%), and lactate (18.8%) (Table 4). However, the carbon flux was altered through repression of the acetate and formate production pathways in IAM1183-CDE/N compared with IAM1183-CDE. The production of each of acetate (71.2%), formate (133%), and 2,3-butanediol (33.9%) was significantly elevated in IAM1183/RN compared with IAM1183 (Table 4), indicating that theses mutations could substantially influence the production of acetate and formate.

The anaerobic metabolic distribution of one mole glucose from adjusting by NadE was also further analyzed to detect effect on IAM1183 in Fig. 6. As shown in Fig. 6, the overexpression of NadE caused different synthesis level of metabolites in IAM1183. It demonstrated that NadE could alter the genes that control the redox state of anaerobic metabolism and therefore promote the anaerobic reduction of terminal electron acceptor hydrogen proton(H^+^) for the hydrogen evolution under anaerobic conditions.

### Detection of specific enzyme activities in mutants

**Fig. 7:**
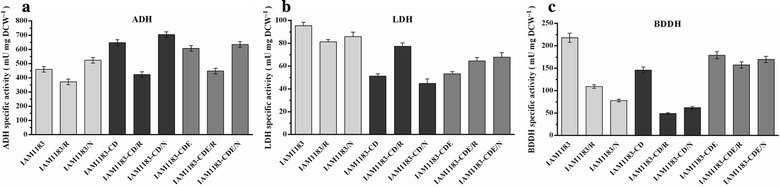
Comparison of enzyme activities between the wild-type strain and mutants (*n* = 3). **a** Alcohol dehydrogenase (EC 1.1.1.1, ADH). **b** Lactate dehydrogenase (EC 1.1.1.27, LDH). **c** 2,3-butanediol dehydrogenase (EC1.1.1.4, BD DH)

To examine how disruption of *nuoCDE*, NadE, or RyhB affected the activities of key oxidoreductases involved in the anaerobic metabolism of all mutants, we compared three specific enzyme activities for which NADH is as cofactor (LDH, EC 1.1.1.27; ADH, EC 1.1.1.1; BDDH, EC 1.1.1.76) among the mutant strains after cultivation for 8 h (mid-exponential phase). Compared with IAM1183, IAM1183-CD exhibited a 1.9-fold and a 1.5-fold decrease in LDH and BDDH activity, respectively, although ADH activity increased by ~ 40% (Fig. 7). The three mutants IAM1183/R, IAM1183-CD/R, and IAM1183-CDE/R exhibited a dramatic reduction in all three enzyme activities. A previous study reported that *hyaA* encoded small subunit of hydrogenase 1, which plays a vital role on hydrogenase 1, was downregulated upon RyhB overproduction under anaerobic conditions [21]. This may underlie the observed decrease in BDDH, LDH, and ADH activities in IAM1183/R, IAM1183-CD/R, and IAM1183-CDE/R.

### Hydrogen production by IAM1183-CDE/N in a fermenter

**Fig. 8:**
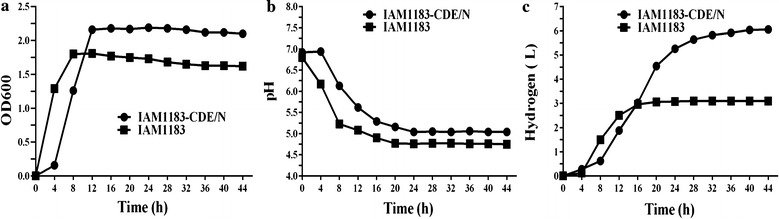
Characterization of IAM1183-CDE/N in a 5-L fermenter. **a** Growth curves. **b** Change in pH. **c** H_2_ evolved during the cultivation between IAM1183-CDE/N and the wild-type strain

Among all mutants, IAM1183-CDE/N produced the most H_2_ (2.39 mol H_2_/mol glucose) during batch serum-bottle fermentation (Table 4), and so we used IAM1183-CDE/N for our pilot large-scale fermentation experiment. A scaled-up fermentation was thus carried out in 3-L glucose-containing medium in a 5-L fermenter. Compared with IAM1183, IAM1183-CDE/N exhibited a prolonged exponential phase (4 h longer) and had 1.3-fold increased cell density based on final OD_600_ after cultivation for 44 h (Fig. 8a). The final pH values of the medium for IAM1183-CDE/N and IAM1183 were 5.04 and 4.82 (Fig. 8b), respectively. These observations indicated that strain IAM1183-CDE/N produced less formate so that more hydrogen produced from formate pathway than IAM1183-CDE or that certain acidic metabolites might have been taken up by the IAM1183-CDE/N cells and utilized as substrates for anabolic processes (Table 5). We however, note that this difference in pH units, i.e., 0.22, is seemingly minor unless this aspect was repeatedly observed in several 5-L fermenter experiments. More glucose was consumed by IAM1183-CDE/N during fermentation, which resulted in a nearly twofold greater production of H_2_ compared with IAM1183 (Fig. 8c).

**Table 5:** Analysis of consumed substrate and anaerobic metabolites after 44 h of cultivation of IAM1183-CDE/N and wild-type IAM1183 in 5-L fermenter

End product	IAM1183	IAM1183-CDE/N	Change trend	Percent change (%)
Glucose consumption (%)	79.47	97.43	↑	22.26
Residual sugar (g/L)	3.08	0.33	↓	87.34
Hydrogen (H_2_) (L)	3.110	6.060	↑	94.85
Succinate (mM)	1.02	3.16	↑	209.8
Lactate (mM)	24.78	26.12	↑	5.400
Acetate (mM)	15.33	11.47	↓	25.17
Formate (mM)	1.260	0.790	↓	37.30
Ethanol (mM)	10.54	12.28	↑	16.50
Citric acid (mM)	1.930	1.750	↓	9.330
Acetoin (mM)	20.73	18.58	↓	10.37
2,3-Butanediol (mM)	6.380	7.740	↑	21.31

After 44 h of culture in the fermenter, the final concentrations of the major excreted metabolites of IAM1183-CDE/N and IAM1183 were also accordingly measured and summarized in Table 5. As shown in Table 5, the final concentrations of lactate and 2,3-butanediol were greater for IAM1183-CDE/N than for IAM1183, whereas the concentrations of acetate and formate decreased; these results were similar to those obtained with the serum-bottle batch system. The mutants generated in this work can be used for further studies of the mechanism of hydrogen metabolism in *E. aerogenes* and for potential applications in biohydrogen production.

## Conclusions

The present work demonstrates that the CRISPR-Cas9-mediated knockout of *nuoCDE* to downregulate NADH hydrogenase in *E. aerogenes* can significantly improve hydrogen yield using glucose as substrate. Moreover, the increasing range of H_2_ production via the NADH pathway was apparently greater than that via formate pathway. The most interesting result was that *nuoE* had a greater influence on *E. aerogenes* in terms of hydrogen metabolism. We also explored the significance of enhancing overall hydrogen production by ∆*nuoCDE* mutants and the influence on the formation of hydrogen production by overexpressing RyhB in *E. aerogene*. Metabolic flux analysis indicated that RyhB could block the alcohol pathway and thereby constitutes a competitive route for hydrogen production. Heterologous expression of the *nadE* gene from *K. pneumonias* in *E. aerogenes* and its ∆*nuoCDE* mutants led to a further increase in hydrogen production and metabolic flux redistribution. Therefore, the small RNA RyhB and NadE may play global regulatory roles in anaerobic metabolism for improved biohydrogen production. The volume of hydrogen produced by the mutant strain IAM1183-CDE/N in the fermenter was almost two times greater than that produced by the wild-type strain. Hence, IAM1183-CDE/N may be a promising candidate to further improve hydrogen production using genetic technologies. The findings reported herein also have the potential for further studies of the mechanism underlying hydrogen metabolism in *E. aerogenes* and for applications to biohydrogen production.

## Additional file

**Additional file 1. Figure S1. ** SDS-PAGE verification of NAD symhetase overexpression in IAM1183 and IAM1183/N. **Figure S2**. Comparison of the control strain IAM1183 and IAM1183/R carrying pKK102-ryhB-cm plasmid by northern blot analysis.

## Abbreviations

NADH: reduced nicotinamide adenine dinucleotide
NadE: nicotinamide adenine dikaryotic synthetase
NAD+: nicotinamide adenine dikaryotic acid
CRISPR/Cas9: clustered regularly interspaced short palindromic repeats/CRISPR-associated
LDH: lactate dehydrogenase
ADH: alcohol dehydrogenase
BDDH: 2,3-butanediol dehydrogenase
PTFE: polytetra fluoroethylene
HPLC: high-performance liquid chromatography
pH: pondus hydrogenii
