# Supplementary material for: Impairment of NADH dehydrogenase and regulation of anaerobic metabolism by the small RNA RyhB and NadE for improved biohydrogen production in Enterobacter aerogenes
Source: Biotechnol Biofuels. 2017 Oct 30;10:248. doi: 10.1186/s13068-017-0938-2 (PMC5663082; doi:10.1186/s13068-017-0938-2)
Supplement: Supplementary file 1 — Additional file 1. Figure S1. SDS-PAGE verification of NAD symhetase overexpression in IAM1183 and IAM1183/N. Figure S2. Comparison of the control strain IAM1183 and IAM1183/R carrying pKK102-ryhB-cm plasmid by northern blot analysis. [file 13068_2017_938_MOESM1_ESM.docx]

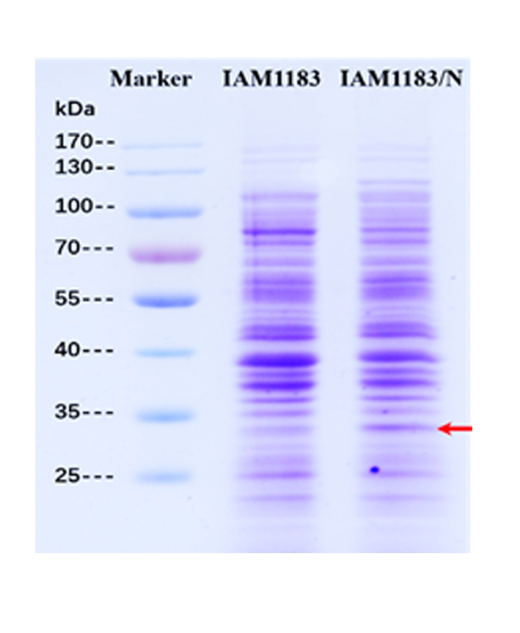


**Fig. S1** SDS-PAGE verification of NAD symhetase overexpression. lane 1 is protein marker; lane 2 is control strain IAM1183; lane 3 is IAM1183/N carrying pET-28a-nadE plasmid.


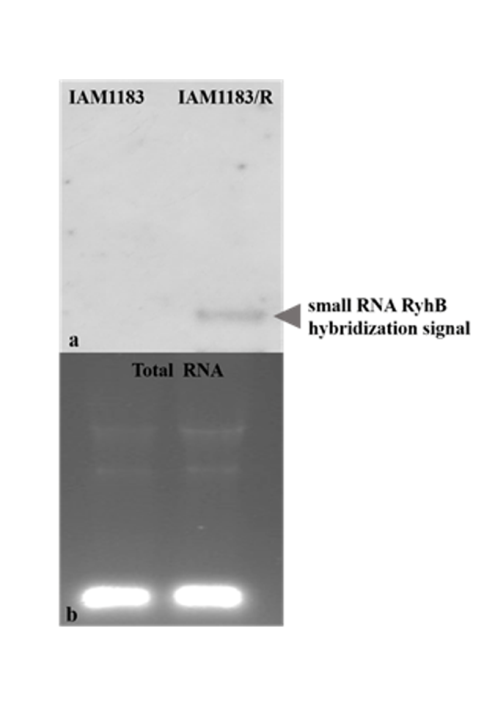


**Fig. S2** Comparison of the control strain IAM1183 (lane 1) and IAM1183/R carrying pKK102-ryhB-cm plasmid (lane 2) by northern blot analysis. **a** Small RNA RyhB hybridization signal of northern blot analysis. **b** Total RNAs (4 ug/sample) from IAM1183 and IAM1183/R.
